# Ethics in writing: Learning to stay away from plagiarism and scientific misconduct

**DOI:** 10.4103/0970-2113.80337

**Published:** 2011

**Authors:** Bharat Bhushan Sharma, Virendra Singh

**Affiliations:** *Division of Allergy and Pulmonary Medicine, Department of Medicine, SMS Medical College, Jaipur, India*

**Keywords:** Ethics, plagiarism, research, scientific misconduct, writing

## Abstract

Fraudulent data and plagiarized text may corrupt scientific medical literature and ultimately harm patients. By prescribing erroneous treatment to an individual, only single patient is affected; but by presenting incorrect data or transcripts, the whole scientific medical universe is affected. Although both scenarios are highly undesirable, one can assume the magnitude of the effect of latter. Writers of scientific medical literature have been found to be involved in plagiarism and other publication misconducts from time to time irrespective of social, economic and geographic structure. The reason of such behavior is not usually obvious. Easy availability of personal computers has led to widespread dissemination of medical literature. As a result, young scientists are now publishing their research more frequently and efficiently. At the same time, this has increased the tendency to submit hurriedly prepared, poorly drafted and even illegitimate publications. Use of some amount of copy–paste followed by modifications during preparation of a manuscript seems to be common. Therefore, the researchers, especially postgraduate students, should be educated continuously about ethical medical writing.

## WHAT IS PLAGIARISM AND SCIENTIFIC MISCONDUCT?

Although plagiarism is difficult to define in a few sentences, it can be viewed as the stealing of another person’s ideas, methods, results or words without giving proper attribution.[1]

Plagiarism usually involves the use of writings belonging to others. The term can be applied to copying of part of own previous published study by a scientist without appropriate citation.[2] Such self-plagiarism is not tolerable in academic writing because authors are supposed to mention closely related previous work in appropriate manner.[3] The work already published by an author becomes a property of scientific medical literature in actual sense and cannot be duplicated.

Use of sentence/s from published medical literature with minor modification in word structure without attribution is also plagiarism. One must be aware of the fact that using published photos or images without written permission is also considered as plagiarism. Easy availability of private computers has led to increase in tendency to use of copy and paste method of writing by young authors.[4] The need of adequate referencing and apprehension of plagiarism raises the important issue about what one requires to cite.[5] In general, any statement that contains a fact that is not universally known or contains factual details should be referenced.

Scientific misconduct (fabrication and falsification of data) is now beginning to be considered similar to other criminal offences and often committed by the same offender.[6] In a meta-analysis by Fanelli *et al*., it was found that medical researchers reported misconduct more frequently than respondents in other fields.[7]

## HOW TO DIAGNOSE PLAGIARISM?

Most of the time, plagiarism is an unintended behavior. But plagiarism can tarnish the image of an author very badly. Reputed journals consider plagiarism as a highly unethical practice and they strongly depreciate such behaviors. Concern about plagiarism in the international community has led to the development of guidelines by Committee On Publication Ethics (COPE).[8] Plagiarism is difficult to detect and poses significant threat to the health of scientific literature. Mostly, the plagiarism is suspected by knowledgeable reviewers and their expertise in a particular field helps them catch subtle defects easily. Editorial staff uses electronic plagiarism-checks to detect plagiarism. The suspicious areas indicated by such tools are then compared carefully by placing both articles in parallel. Abstract similarity is a useful method to detect replication, but comparison of full text article is more helpful.[9] The manual assessment of full text is very laborious but it is highly specific and enables efficient verification.[10]

## PLAGIARISM DIAGNOSED IN MANUSCRIPTS SUBMITTED TO *LUNG INDIA*

We are quite fortunate in having low occurrence of plagiarism in the submitted manuscripts. However, a few manuscripts have been found to contain significant plagiarism. Following are examples of plagiarism in some of the submitted articles to *Lung India* during last year.

### Review Articles

The review articles were routinely scanned for plagiarism.

- In a review article on apoptosis and lung cancer, many sentences were copied from multiple sources, mainly the Wikipedia. Even the tables and images were copied from other websites without written permission or attribution.
- In another review article, significant amount of the copied text from multiple sources was found.

### Original Articles

The original articles were screened for plagiarism only when the reviewers or editors suspected plagiarism based on various features like discrepancy in writing style or font, etc.

Discussion in an original article was copied from a published article with only minor modifications.

An original article was submitted as a part of a study which was already published elsewhere. Published article and manuscript had some overlapping features and mentioned different prevalence of the same disease in the same study population.

Another original article was submitted as part of a study which was already published. Number of patients in the published article was 100. Number of patients in the submitted article was 300. But the percentage of the observed demographic and other parameters was same. Even the *P* values mentioned for different parameters of these 300 patients were exactly the same as that of 100 patients in the published study.

One original article was under peer review process. The peer review process is double blind in the sense that both author and reviewer are not aware of the identity of each other. One reviewer from Bangkok had asked for some modifications. The author in reply copy-pasted some of the contents of published article of the same reviewer, which was subsequently caught by the reviewer himself.

### Case Reports

- An author submitted a manuscript which was regrettably copied word to word from a recently published article of the reviewer.
- One case report was submitted from the USA, which was already published and available on the hospital website.
- One manuscript had been accepted by two reviewers. But due to uncertainty about quality, it was sent to Section Editor for opinion. The Section Editor found out that the introduction and discussion part of the manuscript were copied word to word from a published article.
- Recently, a complete case report including the case history contained copied text. It seemed that the author first copy–pasted complete published article in the document. Then, minor modifications were made to fill the important findings.

### Letter to Editor

This is a small manuscript and usually not screened for plagiarism. But some of the letters submitted to *Lung India* also contained copy–pasted material.

## FROM THE ABOVE EXAMPLES, WHAT ARE THE MOST SERIOUS FORMS OF MISCONDUCTS?

**Figure 1:**
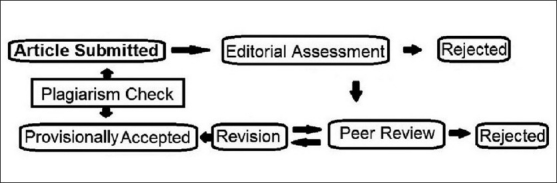
Plagiarism checking policy now adopted by Lung India

Fabrication of data or duplication of article is obviously the most serious form of misconduct. Currently, we consider this as an issue of main concern. The COPE has given clear guidelines on matters like this.[11] An editorial decision was taken to screen all the articles during initial submission and at pre-acceptance phase [Figure 1].

Based on COPE guidelines, the following was the consensus opinion of honorable members of Editorial Board of *Lung India*:

### A. Minor copy–paste in a submitted manuscript

The author should modify the copied text or minor discrepancy in the data.

### B. Considerable copy–paste, copyright violation of images or a table in a submitted manuscript

Manuscript will be sent back for modifications with a communication to author regarding position of journal and disapproval of plagiarism. The author should explain with valid reasons for the mistake, send desired data and assure that no further mistake will be made by him or co-authors in the future.

### C. Extensive copy–paste or fabrication of data/duplication in a submitted manuscript

The manuscript will be rejected. An explanation from author will be demanded. In case of unsatisfactory reply or failure to respond, the head of authors’ institute will be informed. The author/co-authors will be barred from publication in *Lung India* for a period of 2 years.

## DO YOU BELIEVE THAT INEXPERIENCED AUTHORS COMMIT PLAGIARISM?

It may be true to a certain extent, but almost all manuscripts shown above were submitted by senior authors. It also may be speculated that senior scientists depend largely on their junior counterparts for writing of research papers for publication. It may be lack of availability of time for writing or perhaps the need of publishing many articles in a short time period that leads to plagiarism or falsification.

The manuscripts containing plagiarized text and fraudulent data not only distort scientific records but also may harm patients.[12] The editors and referees usually are contended with finding out and rejecting manuscripts containing extensive plagiarism, but something more needs to be done.[13] We should therefore educate young scientists about plagiarism and other publication misconducts. The reason of plagiarism by students may be lack of awareness on appropriate referencing and lack of knowledge on what constitutes plagiarism.[14] As a young scientific writer, it is your responsibility to be very careful during preparation of manuscripts and revisions. You can avoid plagiarism and improve the quality of manuscript by using the following tips:

- Allot sufficient time for writing even if it is a protocol of a study.
- Collect hard copies of all the relevant references.
- Read all the references carefully and highlight important areas.
- Place sufficient attribution while using ideas of others.
- Lines with factual details are to be referenced.
- Reconfirm and decide about appropriateness of inserting references.
- For figures copied from other sources, take written permission.
- Write down all the text by yourself in your own language.
- Never use copy and paste while writing. If you are not good at typing, you can take the help of a typist or a companion.
- Before submitting an article, make sure that you have prepared all the files, figures and references according to the journal’s instructions.

Statement about ethical medical practice – “Good human acts fulfill human needs in a balanced manner, bad acts do not”[15] – applies to medical research and its reporting as well.
